# Association of central capillary refill time with mortality in adult trauma patients: a secondary analysis of the crash-2 randomised controlled trial data

**DOI:** 10.1186/s13049-025-01407-1

**Published:** 2025-05-12

**Authors:** Sabrina Jegerlehner, Tim Harris, Martin Mueller, Ben Bloom

**Affiliations:** 1 Department of Internal and Emergency Medicine, Buergerspital Solothurn, Schoengruenstrasse 42, 4500 Solothurn, Switzerland; 2 Department of Emergency Medicine Inselspital Bern, University Hospital, Freiburgstrasse 16 C, 3010 Bern, Switzerland; 3 Blizzard Institute, Queen Mary University London, 327 Mile End Road, London, UK; 4 Emergency Department, Royal London Hospital, Barts Health NHS Trust, London, UK; 5 Centre for Trauma Science, Blizzard Institute, Queen Mary University London, London, UK

**Keywords:** Emergency medicine, Central capillary refill time, Trauma, Mortality, Shock, Pre-hospital

## Abstract

**Background:**

Trauma-related injuries account for up to 4.4 million deaths annually worldwide. Failure to identify haemorrhage in trauma patients increases mortality. This study examines the association of central capillary refill time (CRT) and mortality in adult trauma patients, especially in the subgroup with normal heart rate (HR) and blood pressure (BP).

**Methods:**

This retrospective observational study analysed data from the CRASH-2 trial, conducted in 274 hospitals across 40 countries and 5 continents between May 2005 and January 2010. A total of 19,054 out of 20,207 adult trauma patients with recorded CRT and complete dataset were included. CRT was taken centrally (sternum) and categorized as ≤ 2, 3–4, and ≥ 5 s. The primary outcome was 28-day mortality, while secondary outcomes included need for transfusion, surgical intervention and thromboembolic events. Univariable and multivariable logistic regression analysis were conducted, incorporating random effects for continent/cluster. Receiver operating characteristic curves were used to assess the discriminatory ability of central CRT measurement.

**Results:**

Among the patients, 6,756 (35.5%) had a CRT ≤ 2 s, 9,142 (48%) had a CRT of 3–4 s, and 3,156 (16.6%) had a CRT ≥ 5 s. Compared to the reference category (CRT ≤ 2 s), the odds of death were significantly higher in patients with CRT of 3–4 s (OR 1.7, 95% CI 1.6–1.9) and CRT ≥ 5 s (OR 3.2, 95% CI 2.8–3.5). Higher CRT was also associated with an increased likelihood of blood transfusion, surgical intervention, and thromboembolic events. The AUC values ranged from 0.63 to 0.74 and were consistent with a significant association between the variables.

**Conclusion:**

Central CRT is associated with increased mortality and adverse outcomes in trauma patients. In bleeding trauma patients, an increasing central CRT is linked to higher mortality risk, with a central CRT ≥ 5 s being particularly predictive of worse outcomes. This also applies to patients with stable vital signs (normal HR and BP), suggesting that CRT may offer additional value as an indicator of hidden hypoperfusion.

**Supplementary Information:**

The online version contains supplementary material available at 10.1186/s13049-025-01407-1.

## Background

Injuries, whether they result from accidents or acts of violence, claim the lives of 4.4 million individuals worldwide annually, accounting for nearly 8% of the total global mortality rate [1]. Haemorrhage accounts for about one third of in-hospital trauma deaths [2], and contributes to other causes of death such as head injury and organ dysfunction [3]. Failure to identify patients with haemorrhage and institute early treatment increases mortality [4]. However, estimation of the extent of haemorrhage is difficult, especially in compensated shock when haemodynamic parameters are still within normal range.

CRT is defined as the time taken for a skin capillary bed to regain its colour after sufficient pressure has been applied to cause blanching [5]. Vasoconstriction leading to reduced distal capillary bed perfusion is considered an early compensatory mechanism to shock. Delayed reperfusion is a marker of circulatory impairment. CRT may reflect alterations in the microcirculation even when systemic haemodynamics remain normal [6]. Therefore, CRT is advocated as a simple and rapid assessment method for (compensated) shock and is commonly used both in-hospital and pre-hospital [7].

CRT is widely used in the assessment of ill neonates and children, e.g. for hypovolemia and sepsis. A CRT of ≤ 2 s is a treatment target for resuscitation in sepsis. Several studies support the use of CRT as a valuable clinical parameter for assessing perfusion and guiding resuscitation in septic shock, offering a non-invasive and readily accessible alternative to traditional markers like lactate [8–10]. CRT is part of the assessment of circulation in the European Resuscitation Council algorithm for pre-hospital care, and is one of five components of the Trauma Score [6, 7, 11–14]. Its use is advocated by the adult and paediatric life support algorithms. However, there are few outcome data to support the use of CRT in adult trauma patients.

The association of central CRT with mortality in adult trauma patients was investigated, using data from the CRASH-2 trial. The primary objective was to investigate the association of central CRT with 28-day mortality in adult trauma patients, especially in the subgroup with normal heart rate (HR) and blood pressure (BP) to address the value of central CRT to detect hidden hypoperfusion.

## Methods

### Study design

This was a secondary analysis of the CRASH-2 trial data [15]. CRASH-2 has been previously described [16]. Briefly, CRASH-2 was a randomised controlled trial of tranexamic acid versus placebo in adult patients with trauma and significant haemorrhage. It was conducted in 274 hospitals in 40 countries from May 2005 to January 2010 and included 20,211 adult trauma patients [15].

### Inclusion and exclusion criteria

The CRASH-2 trial included adult (age ≥ 16 years) trauma patients with significant haemorrhage (SBP < 90 mmHg and/or HR > 110 beats per minute), or who were considered to be at risk of significant haemorrhage, and who were within eight hours of the injury. Patients for whom the responsible doctor considered there was a clear indication for or a clear contraindication against antifibrinolytic therapy were not randomly assigned. There were no other pre-specified exclusion criteria in the CRASH-2 trial.

### Data extraction methods

In the original trial, 20,211 patients were randomly assigned to receive TXA or placebo. After exclusion of four patients who withdrew consent and 80 patients who were lost to follow-up, 20,127 patients were included in the intention-to-treat analysis. For the present study, we screened the records of the 20,207 patients who provided informed consent and excluded those with missing data on capillary refill time.

### Variable definitions

CRT was defined as the time (in seconds) taken for a skin capillary bed to regain its colour after sufficient pressure has been applied to cause blanching. CRT was measured centrally on the middle of the chest once as part of the initial assessment at the time of randomisation based on local clinical practice. In the CRASH-2 trial, central CRT was collected in seconds. For this study, central CRT was categorised as ≤ 2, 3–4, and ≥ 5 s based on the original structure of the CRASH-2 dataset.

In this trauma patient population, normal BP was defined as systolic blood pressure (SBP) ≥ 100 mmHg and normal HR was defined as ≤ 100/minute. Shock was defined as SBP < 90 mmHg and HR > 110/min.

### Primary and secondary outcomes

The primary outcome was 28-day mortality of any cause after the traumatic injury. Patients discharged alive before 28 days were assumed to be alive at 28 days unless there was evidence otherwise. Secondary outcomes were transfusion (defined as the administration of any blood product, including packed red blood cells, whole blood, plasma, or platelets), need for surgical intervention (defined as any operative procedure performed to control bleeding after randomization, and occurrence of VTE (defined as the occurrence of either deep vein thrombosis or pulmonary embolism diagnosed during the hospital stay based on clinical suspicion and confirmed by imaging).

### Data analysis

Statistical analysis was conducted using STATA 18.0 (MP, StataCorp, College Station, TX, USA). Statistical significance was defined as *p* value < 0.05.

For the continuous variable we estimated means and standard deviations (SD) or median and interquartile range (IQR) for normally and non-normally distributed data respectively based on Shapiro–Wilk testing. Categorical data were reported as proportions. Differences in continuous data were analysed using the *t* test (normal distribution) or Wilcoxon rank sum test (non-normal distribution); categorial data were compared using the *chi*^*2*^ test.

For the primary outcome and all other binary outcomes, odds ratios (ORs), 95% confidence intervals (CIs) and two-sided *p* values for statistical significance were calculated. Univariable and multivariable logistic regression analysis was performed to assess the association of CRT with mortality and all other secondary outcomes. Because patients from different countries represent a nested (clustered) dataset, random effect modelling for countries was used to account for the inter-country variance (clustering). Covariates were selected based on theoretical considerations and included age group, type of injury (blunt, penetrating, combined), and administration of TXA.

Given that the upper reference limit for CRT is typically ≤ 2 s in children [14, 17–20], and recent studies in emergency and critical care have defined the normal CRT value for adults as ≤ 3 s, [21, 22] we conducted sensitivity analyses using the ≤ 3 s threshold. Additionally, CRT was analysed as a continuous parameter using a restricted cubic spline regression with a mixed-effects (random effect for country) generalized linear model with Bernoulli family and logit link function.

Receiver operating characteristic (ROC) curves were used to assess the discrimination (C statistic, area-under-the-curve [AUC]) of the logistic regression model [23].

## Results

**Fig. 1:**
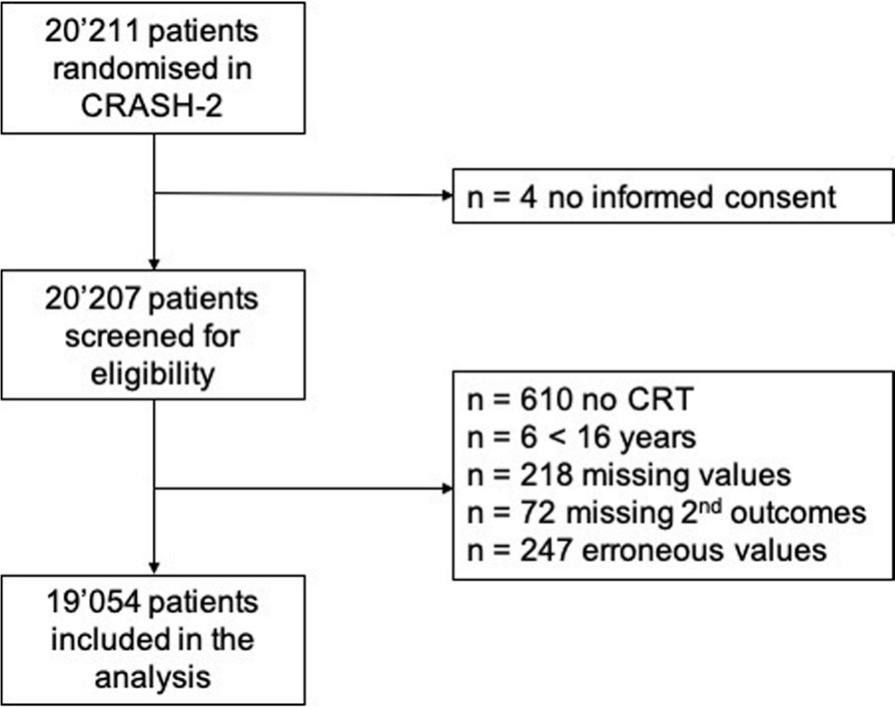
Study overview

Overall, 20,207 patients were included in the original dataset, of whom 611 (4%) did not have a CRT recorded and 543 (3%) had missing or erroneous values and were therefore excluded. The final dataset analysed included 19,054 patients (Fig. 1). Baseline characteristics of the study population are shown in Table 1. 16,000 (84%) patients were male, the median age was 31 (IQR 24–43) years, 10,562 (55%) suffered from blunt trauma and 6,179 (32%) from penetrating trauma. 6,756 (36%) patients had a CRT ≤ 2 s, 9,142 (48%) from 3–4 s, and 3,356 (17%) ≥ 5 s. Over the study period of 28 days, 2,802 (15%) patients died, of which 1,154 (6%) died from a head injury, 902 (5%) died from haemorrhage, and 80 (< 1%) patients died from a thromboembolic event.

**Table 1:** Baseline characteristics of the study patients

	**Total *n* = 19,054**		**Survived *n* = 16,252**		**Death *n* = 2801**		***p* value**
**Age (years)**	31	[24; 43]	30	[23; 42]	35	[25; 48]	< 0.001
16–40 years	13,716	[72.0]	11,929	[73.4]	1,787	[63.8]	
41–65 years	4,575	[24.0]	3,785	[23.3]	790	[28.2]	
66–80 years	674	[3.5]	489	[3.0]	185	[6.6]	
> 80 years	89	[0.5]	49	[0.3]	40	[1.4]	< 0.001
**Gender**							
Male	16,000	[84.0]	13,656	[84.0]	2,344	[83.7]	
Female	3,054	[16.0]	2,596	[16.0]	458	[16.3]	0.620
**Type of injury**							
Blunt	10,562	[55.4]	8,794	[54.1]	1,768	[63.1]	
Penetrating	6,179	[32.4]	5,567	[34.3]	612	[21.8]	
Combined	2,313	[12.1]	1,891	[11.6]	422	[15.1]	< 0.001
**Vital parameters**							
SBP (mmHg)	96	[80; 110]	100	[86; 111]	90	[70; 100]	< 0.001
HR (per minute)	105	[90; 120]	102	[90; 118]	110	[96; 126]	< 0.001
RR (per minute)	22	[20; 26]	22	[20; 26]	24	[20; 30]	< 0.001
Shock	3,077	[16.1]	2,242	[13.8]	835	[29.8]	< 0.001
**CRT groups**							
0–2s	6756	[35.5]	6069	[37.3]	687	[24.5]	
> 2–4s	9142	[48.0]	7772	[47.8]	1,370	[48.9]	
> 4s	3156	[16.6]	2411	[14.8]	745	[26.6]	< 0.001
**28-day mortality**							
Survived	16,252	[85.3]	16,252	[100.0]	0	[0.0]	
Dead	2,802	[14.7]	0	[0.0]	2,802	[100.0]	< 0.001
**Cause of death**							
Bleed	902	[4.7]	n.a		902	[32.2]	
Head injury	1,154	[6.1]	n.a		1,154	[41.2]	
VTE	80	[0.4]	n.a		80	[2.9]	
Other	666	[3.5]	n.a		666	[23.8]	< 0.001
**Secondary outcomes**							
Transfusion	9,534	[50.0]	7,702	[47.4]	1,832	[65.4]	< 0.001
Surgery	9,580	[50.3]	8,139	[50.1]	1,441	[51.4]	0.188
VTE	334	[1.8]	193	[1.2]	141	[5.0]	< 0.001

*CRT* central capillary refill time, *HR* heart rate, *N* absolute count, *RR* respiratory rate, *s* seconds, *SBP* systolic blood pressure, Surgery: need for surgical intervention, *VTE* thromboembolic event

In multivariable logistic regression analysis, the OR for death was 1.76 (95% CI 1.58–1.95) and 2.32 (95% CI 2.83–3.65) in the CRT categories of 3–4 s and ≥ 5 s respectively compared to the reference category of ≤ 2 s (Table 2).

**Table 2:** Multivariable logistic regression analysis: OR for 28-day mortality by CRT category against reference category (CRT ≤ 2 s)

**CRT group**	**OR death all**	**95% CI**	***p* value**	**AUC**
0–2 s	1			
> 2–4 s	1.76	1.58–1.95	< 0.001	
> 4 s	3.21	2.83–3.65	< 0.001	0.698
**CRT group**	**OR death in shock**			
0–2 s	1			
> 2–4 s	1.47	1.15–1.88	0.002	
> 4 s	2.43	1.86–3.18	< 0.001	0.706
**CRT group**	**OR death no shock**			
0–2 s	1			
> 2–4 s	1.78	1.58–2.00	< 0.001	
> 4 s	2.69	2.30–3.13	< 0.001	0.703
**CRT group**	**OR transfusion**			
0–2 s	1			
> 2–4 s	1.39	1.30–1.49	< 0.001	
> 4 s	2.06	1.88–2.27	< 0.001	0.663
**CRT group**	**OR surgery**			
0–2 s	1			
> 2–4 s	1.25	1.16–1.34	< 0.001	
> 4 s	1.57	1.42–1.73	< 0.001	0.696
**CRT group**	**OR VTE**			
0–2 s	1			
> 2–4 s	1.57	1.16–2.11	< 0.001	
> 4 s	2.45	1.76–3.43	< 0.001	0.739

*AUC* area under the curve, *CI* confidence interval, *CRT* capillary refill time, *OR* odds ratio, *s* seconds, surgery: need for surgical intervention, *VTE* venous thromboembolic event

**Fig. 2:**
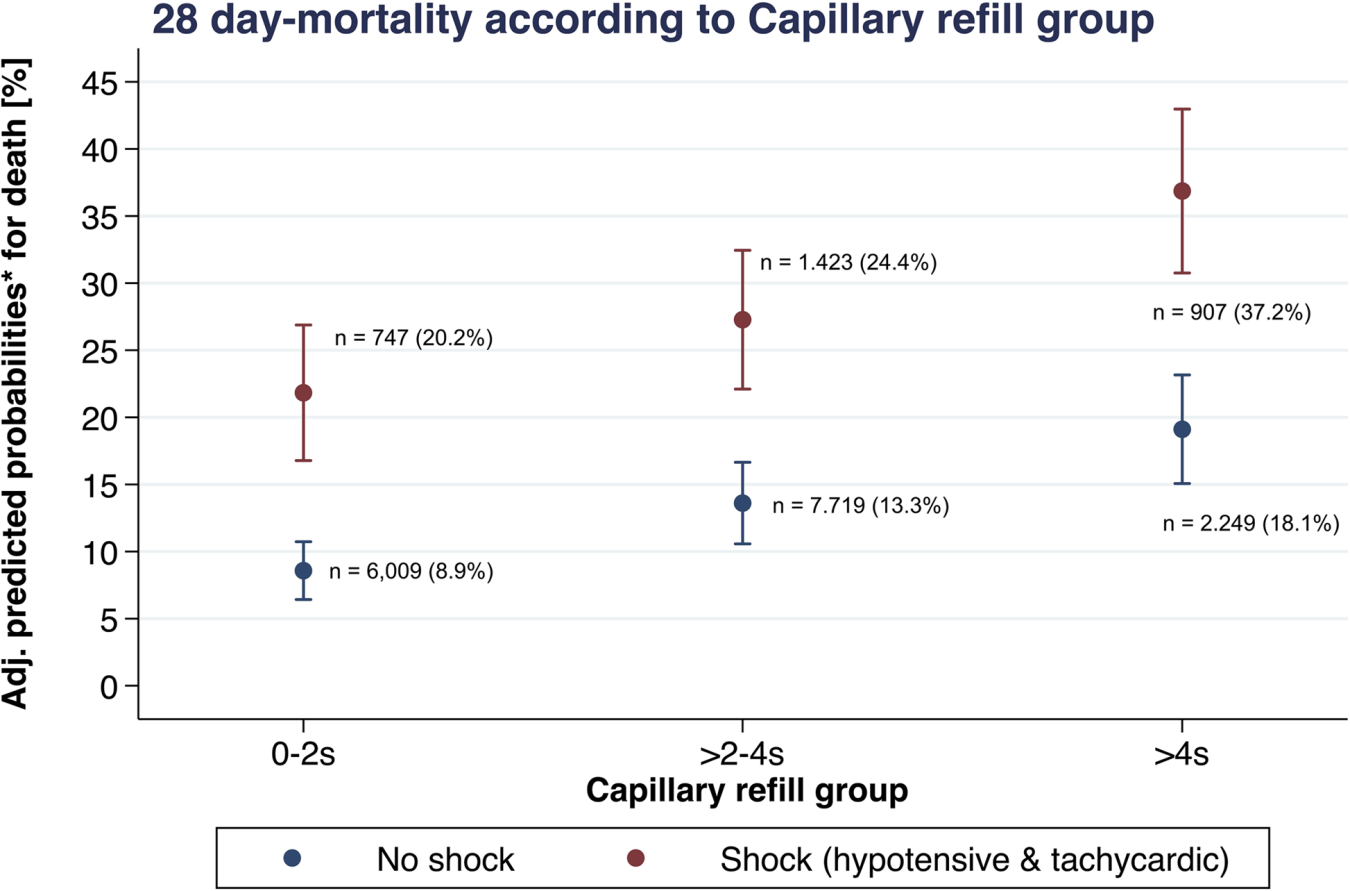
OR for death in different CRT groups depending on haemodynamic parameters (shock versus no shock). CRT: central capillary refill time, CI: confidence interval, OR: odds ratio

In the subgroup of patients with normal HR and BP, i.e., “no shock”, there was an OR for death of 1.78 (95% CI 1.58–2.00) and 2.69 (95% CI 2.30–3.13) in the CRT categories of 3–4 s and ≥ 5 s respectively (Table 2 and Fig. 2).

Secondary outcomes shown in Table 2. An increasing CRT was associated with increased risk of transfusion, surgical intervention, and occurrence of thromboembolic events with OR between 1.25–1.57 for CRT of 3–4 s and OR of 1.57–2.45 for CRT of ≥ 5 s.

### Sensitivity analyses

The analysis was repeated using an upper reference range of ≤ 3 s. The OR for death was 2.37 (95% CI 2.16–2.60) in the CRT category of ≥ 3 s, and the OR for all outcomes were also higher as compared to the ≤ 2 s threshold (Table S1).

**Fig. 3:**
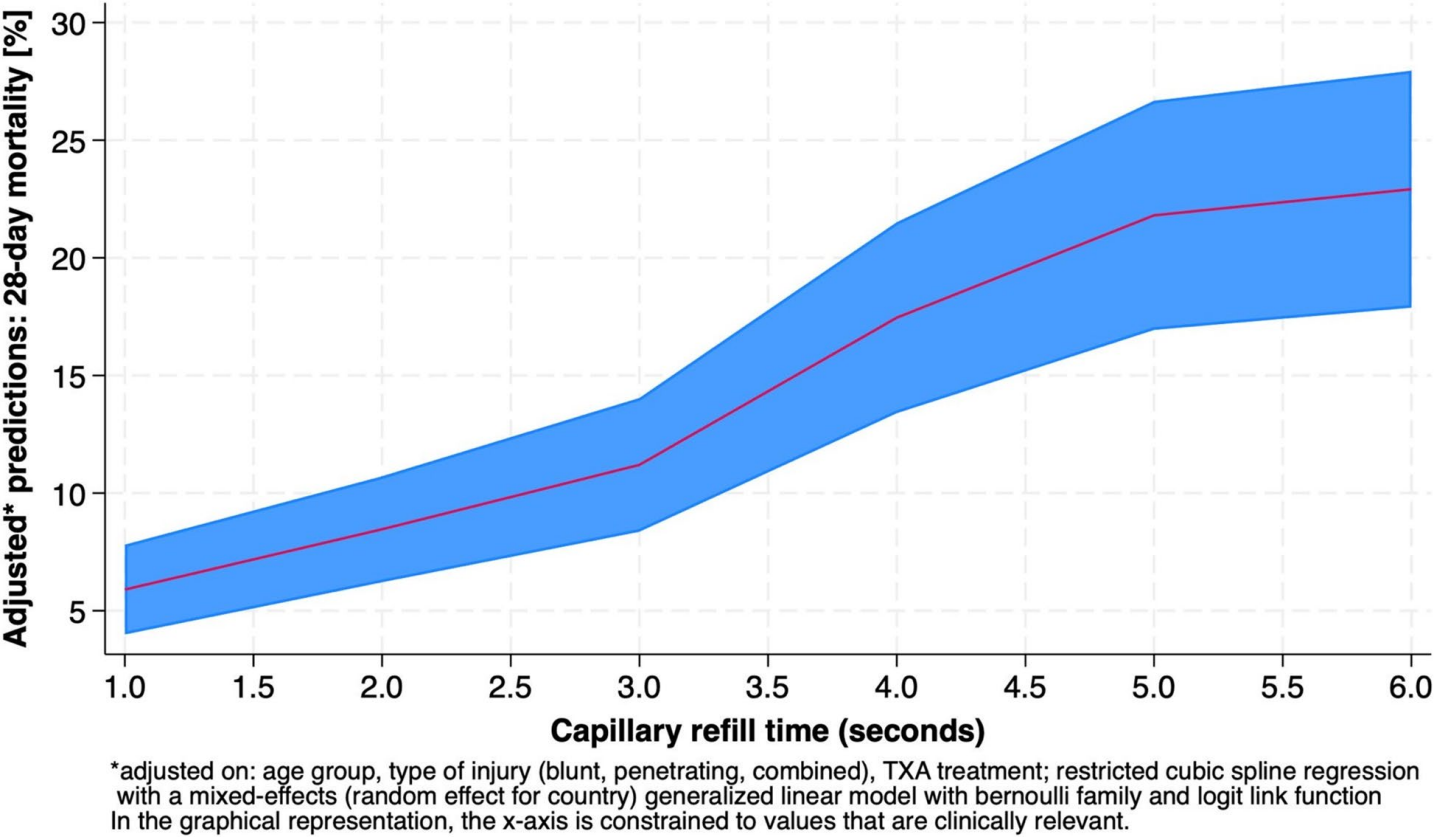
Sensitivity analysis: CRT as continuous variable. Restricted cubic spine regression with a mixed-effects (random effect for country) generalized linear model. *Adjusted on age group, type of injury, TXA treatment. CRT: central capillary refill time, TXA: tranexamic acid

Additionally, CRT was analysed as continuous variable. Longer CRT was associated with higher adjusted 28-day mortality. Predicted mortality rose from about 7% at 1 s to 25% at 6 s. The association was approximately linear through 5 s, with greater uncertainty at longer CRT (Fig. 3).

## Discussion

In this study using data from nearly 20,000 trauma patients, increasing central CRT was associated with an increased risk of death overall and in subgroups of patients with normal HR and BP. In patients with central CRT ≥ 5 s, there was greater than three-fold risk for death, and a more than twofold risk of other adverse outcomes such as need for transfusion and occurrence of thromboembolic events.

### Comparisons with other studies

Jouffrouy et al. [18] examined the association of CRT with mortality in patients with septic shock. They found a significant association of CRT values > 4 s with 28 day mortality (OR 1.5, 95% CI 1.0–2.4). However, in contrast to our study, this is a retrospective single centre study, and it included patients in septic shock with an overall mortality of 36%—higher than the mortality of the trauma patients in the CRASH-2 trial. Mrgan et al. [24] performed a prospective cohort study with adult acute medical patients. They found an increasing one-day mortality with abnormal CRT; and a high negative predictive value for death with normal CRT. However, those were again medical patients, and with a median age of 66 years they were substantially older than the patients in CRASH-2. Additionally, Mrgan et al. concluded that other vital signs such as HR had a stronger association with death and were therefore better predictors for mortality than CRT [24]. This is contrary to our results, where even patients with normal HR and BP had an increased risk for death when CRT was prolonged.

The test accuracy of central CRT assessment with ROC AUC values of about 0.7 is acceptable for prediction and even better for association. These results are in contrast with other studies. In their prospective cohort study, Londono et al. [25] examined the association of clinical hypoperfusion parameters such as CRT with lactate levels and mortality. They found that none of the clinical parameters had an adequate discriminatory capacity to detect hyperlactatemia (AUC < 0.62) or to predict mortality; and that only hyperlactatemia was associated with mortality [25].

In contrast to the trials above, CRT was measured centrally in our study, which represents a significant methodological difference. The limitations of CRT as a clinical test are well known with CRT measurements varying with age, gender, temperature, site of assessment and light conditions [20, 26]. Another limitation of CRT measurement is its susceptibility to inter-rater variability [27]; however, this is a common challenge with many bedside tests, which nevertheless remain valuable tools for point-of-care assessment.

### Strengths and limitations

This study is the first to investigate the association of central CRT with mortality in adult trauma patients using comprehensive and exhaustive data from the multicenter randomised controlled CRASH-2 trial.

The CRASH-2 study population is predominantly young and male, and these results may not be applicable in other populations.

Adjustment for external and ambient confounders was not possible in this study. However, central CRT was used, which was standardised across all CRASH-2 trial sites, and which is less dependent on temperature. Skin pigmentation was not specifically recorded, which could contribute to measurement variability in CRT. Nonetheless, the trial’s broad geographic scope—spanning five continents—likely ensured inclusion of a diverse patient population, potentially mitigating the impact of this limitation. Additionally, CRT was recorded according to local clinical practice, which may introduce variability in measurement. While this represents a potential limitation, it also aligns with the pragmatic nature of the CRASH-2 trial and reflects real-world clinical conditions, thereby enhancing the external validity of our findings.

It is also important to note that the association between CRT and mortality may not be entirely attributable to hypoperfusion alone. In patients with traumatic brain injury (TBI), prolonged CRT may reflect the overall severity of trauma or a broader systemic response rather than isolated circulatory compromise.

Furthermore, the CRASH-2 trial included patients who were judged to have or to be at risk of on-going significant haemorrhage. These inclusion criteria are purely clinical and could represent a source of selection bias, meaning that our findings may not be fully generalizable to all trauma patients, especially those with isolated injuries or without major bleeding. However, the large number of trial participants in different healthcare settings, and thus heterogeneity of the study population, still guarantees generalisability of the results.

Patients included in the CRASH-2 trial were required to have a detectable pulse and BP to meet the inclusion criteria, which led to the exclusion of those in traumatic cardiac arrest who only met inclusion criteria after return of spontaneous circulation.

The primary endpoint of the study was 28-day mortality, a widely accepted outcome measure in trauma research. However, this endpoint is relatively distant from the time of injury, and earlier outcome data were not available for analysis, limiting our insight into short-term physiological changes.

This study was not designed prospectively to determine a threshold for central CRT values above which mortality is increased. However, different cut-offs and definitions for abnormal CRT as previously described in the literature were investigated. There was increasing mortality with rising CRT, and therefore CRT is a continuous rather than a binary predictor for mortality. While HR and BP reflect macro-hemodynamics and can appear normal during compensated shock, CRT assesses microcirculatory perfusion, which may be impaired earlier.

Additionally, CRT was measured only once, with no serial assessments available throughout the trial period. This limits our ability to assess temporal trends or responsiveness to treatment over time.

Finally, the CRASH-2 dataset does not include detailed physiological or biochemical parameters such as SOFA scores, urinary output, or lactate levels. Future studies integrating CRT with such markers of organ dysfunction and perfusion may provide a more comprehensive understanding of its prognostic value.

## Conclusion

In bleeding trauma patients, rising central CRT is associated with increased mortality. A central CRT ≥ 5 s is associated with increased mortality in trauma patients, even when HR and BP are within normal range. Thus, prolonged CRT can reveal hidden hypoperfusion, making it a more sensitive marker in certain clinical contexts.

## Supplementary Information

Supplementary Material 1.

## Data Availability

CRASH-2 data is publicly available.

## Abbreviations

AUC: Area under the curve
CRASH: Clinical randomisation of an antifibrinolytic in significant haemorrhage
CRT: Capillary refill time
CI: Confidence Interval
DCRT: Digital CRT
HR: Heart rate
IQR: Interquartile range
Surgery: Need for surgical invervention
OR: Odds ratio
ROC: Receiver operating characteristic curves
RR: Relative risk
SBP: Systolic blood pressure
SD: Standard deviation
SOFA: Sequential organ failure assessment
TBI: Traumatic brain injury
TXA: Tranexamic acid
VTE: Venous thromboembolic event
