# Supplementary material for: Association of central capillary refill time with mortality in adult trauma patients: a secondary analysis of the crash-2 randomised controlled trial data
Source: Scand J Trauma Resusc Emerg Med. 2025 May 12;33:82. doi: 10.1186/s13049-025-01407-1 (PMC12070708; doi:10.1186/s13049-025-01407-1)
Supplement: Supplementary file 1 — Supplementary Material 1. [file 13049_2025_1407_MOESM1_ESM.docx]

**SUPPLEMENTARY MATERIAL**

## **Table S1.** Sensitivity analysis. Multivariate logistic regression analysis with different upper reference limit of CRT (≤ 3 s). OR for 28-day mortality by CRT category against reference category (CRT ≤ 3 s). AUC: area under the curve, CI: confidence interval, CRT: capillary refill time, OR: odds ratio, s: seconds, surgery: need for surgical intervention, VTE: venous thromboembolic event.

| **CRT group** | **OR death all** | **95% CI** | **p value** | **AUC** |
| --- | --- | --- | --- | --- |
| ≤ 3 s | 1 |  |  |  |
| > 3 s | 2.37 | 2.16-2.60 | <0.001 | 0.701 |
| **CRT group** | **OR death in shock** |  |  |  |
| ≤ 3 s | 1 |  |  |  |
| > 3 s | 2.11 | 1.90-2.35 | <0.001 | 0.727 |
| **CRT group** | **OR death no shock** |  |  |  |
| ≤ 3 s | 1 |  |  |  |
| > 3 | 2.10 | 1.88-2.34 | <0.001 | 0.705 |
| **CRT group** | **OR transfusion** |  |  |  |
| ≤ 3 s | 1 |  |  |  |
| > 3 s | 1.8 | 1.68-1.93 | <0.001 | 0.665 |
| **CRT group** | **OR surgery** |  |  |  |
| ≤ 3 s | 1 |  |  |  |
| > 3 s | 1.49 | 1.39-1.59 | <0.001 | 0.698 |
| **CRT group** | **OR VTE** |  |  |  |
| ≤ 3 s | 1 |  |  |  |
| > 3 s | 1.72 | 1.36-2.17 | <0.001 | 0.740 |
